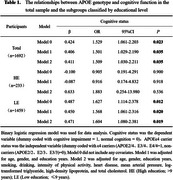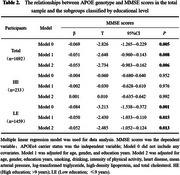# Effects of Apolipoprotein E Genotype and Education on Cognitive Function

**DOI:** 10.1002/alz70860_099578

**Published:** 2025-12-23

**Authors:** Shan Wei, Peijie Liu, Suhang Shang, Liangjun Dang, Ling Gao, Jingyi Wang, Qiumin Qu, Jin Wang

**Affiliations:** ^1^ The First Affiliated Hospital of Xi'an Jiaotong University, Xi'an, Shaanxi, China; ^2^ Huxian Hospital of Traditional Chinese Medicine, Xi'an, Shaanxi, China

## Abstract

**Background:**

To analyze the relationship between APOE genotype and cognitive impairment among individuals aged 40 and above in rural Xi'an, and to explore the potential influence of education on this relationship.

**Method:**

All permanent residents aged 40 and above from two villages in Huyi District, Xi'an City were selected as research subjects, employing a cross‐sectional survey approach. The Mini‐Mental State Examination (MMSE) was utilized to assess overall cognitive function, with MMSE scores below the threshold values (illiterate ≤17, primary school ≤20, junior high and above ≤24) considered as cognitive impairment. Fasting elbow venous blood was drawn in the morning, and the apolipoprotein E (APOE) genotype was determined. The population was divided into low education (LE; ≤9 years) and high education (HE; >9 years) groups based on educational level. Univariate and multivariate analysis were applied to explore the association between APOE genotype and cognitive impairment, as well as MMSE scores in both the total and stratified populations.

**Result:**

Out of 1692 participants, there were 263 APOEε4 carriers (E2/4, E3/4, E4/4) (15.3%), and 205 individuals met the criteria for cognitive impairment (12.1%). Binary logistic regression and multiple linear regression analyses revealed that, in both the total population and the LE population, compared to APOEε4 non‐carriers (E2/2, E2/3, E3/3), APOEε4 carriers exhibited a significantly higher risk of cognitive impairments (total population: OR=1.509, *p* = 0.035; LE: OR=1.604, *p* = 0.019) (Table 1, Model2), and their MMSE scores were significantly lower (total population: β=‐0.053, *p* = 0.006; LE: β=‐0.052, *p* = 0.013) (Table 2, Model2). However, in the HE population, there was no significant difference in the prevalence of cognitive impairment (OR=1.883, *p* = 0.536) (Table 1, Model2) and MMSE scores (β=0.001, *p* = 0.992) (Table 2, Model2) between APOEε4 carriers and non‐carriers.

**Conclusion:**

The APOEε4 allele was associated with an increased risk of cognitive impairment in individuals aged 40 and above in rural areas of Xi'an, while higher educational attainment may offer protective effects against cognitive impairment in APOEε4 carriers.